# Editorial: Raising the bar: advancing therapeutic strategies for fighting communicable and noncommunicable diseases

**DOI:** 10.3389/fphar.2024.1486889

**Published:** 2024-09-17

**Authors:** Dariel Wilbert Tanoto, Jia Wen Lee, Yee Kien Chong, Rafidah Lani, Pouya Hassandarvish, Adrian Oo

**Affiliations:** ^1^ Department of Microbiology and Immunology, Yong Loo Lin School of Medicine, National University Health System, National University of Singapore, Singapore, Singapore; ^2^ Retroviral Immunology, The Francis Crick Institute and Department of Medicine, Faculty of Medicine, Imperial College London, London, United Kingdom; ^3^ Department of Medical Microbiology, Faculty of Medicine, Universiti Malaya, Kuala Lumpur, Malaysia; ^4^ Tropical Infectious Disease Research and Education Centre (TIDREC), Universiti Malaya, Kuala Lumpur, Malaysia; ^5^ Infectious Diseases Translational Programme, Yong Loo Lin School of Medicine, National University of Singapore, Singapore, Singapore

**Keywords:** infectious disease, communicable diseases, therapeutic, virus infection, bacterial infection

Humankind is constantly plagued with invisible yet devastating pathogens. Just a mere 4 years ago, the world came to a standstill as SARS-CoV-2 grew rampant and disrupted every aspect of society. Similarly, other pathogens discussed in this Research Topic can bring about equally detrimental effects should it be left undetected and untreated. Consequently, various authors have presented their findings on how this can be mitigated, one step at a time.

Various researchers have presented findings on pathogens that cause communicable diseases such as the group B streptococcal (GBS) presented by Liu and Ai. Liu and Ai described various factors that allow GBS to circumvent the immune system, such as antibiotic resistance, as well as virulence factors that are common across different bacteria. GBS infection in obstetrics and gynecology highlights its significance as a cause of maternal and neonatal morbidity and mortality (Cho et al., 2022). For women with pre-labor rupture of membranes at term who are GBS-colonized, induction of labor is associated with reduced rates of neonatal infection compared to expectant management (Money and Allen, 2016). These advancements aim to refine prevention strategies and potentially decrease antibiotic use in the future.

Drug-resistant pathogens have been an increasingly concerning public health threat. Yu et al. described the potential side effects of using bedaquiline, which disrupts the *mycobacterium* adenosine triphosphate synthase (Chahine et al., 2014), as a treatment for multidrug-resistant tuberculosis patients. Usage of bedaquiline has been limited due to its adverse association with QTc prolongation, an irregular cardiovascular phenomenon (Pont et al., 2017; Gao et al., 2021). In their research, Yu et al. employed metabolomic analysis on the urine specimens of QTc-prolonged and QTc un-prolonged patients to uncover differences in various biomarkers between the two groups. Eventually, they concluded that two lysophosphatidylethanolamine (LPE) molecules were the most ideal biomarkers with the highest sensitivity and specificity. Their discovery not only potentially allows a more pervasive use of bedaquiline, but also highlights the ease of detecting vital biomarkers through less-invasive methods.

Urine samples may also be used as a variable to improve current drug dosing regimens. Residual diuresis from patients receiving continuous renal replacement therapy (CRRT) may promote drug elimination, consequently affecting the efficacy of drugs administered using the standard dosage, such as vancomycin. Yet, aggressive dosing regimens, frequent monitoring, and individualized approaches based on population pharmacokinetic (PopPK) models are recommended for patients receiving CRRT (Omrani et al., 2015). Yu et al. therefore proposed a PopPK model to guide vancomycin dosing in CRRT patients, incorporating residual diuresis as a factor. They performed the first multicenter retrospective study, measuring daily urine volume as a variable, highlighting the importance of individualized dosing of antibiotics in different patients.

With the progressive use of combinatorial therapies against many pathogens, drug-drug interactions gradually becomes a challenge. In their research, Jacobsson et al. determined the pharmacodynamics of first-in-class zoliflodacin and doxycycline combinatorial therapy against gonorrhea and *chlamydia* coinfections. Using a dynamic *in-vitro* hollow-fiber interaction model (HFIM), Jacobsson et al. observed high efficacy in the combinatorial therapy on *Neisseria gonorrhoeae* compared to a previous static *in-vitro* study (Foerster et al., 2019) and zoliflodacin monotherapy. Zoliflodacin also showed abilities to suppress the emergence of antimicrobial-resistant mutants. Combination therapy with doxycycline proved effective in HFIM, eradicating *N. gonorrhoeae* strains at 3 g zoliflodacin doses without resistance emergence.

On the viral front, the COVID-19 pandemic made therapeutics discovery against viruses even more relevant. Among the earliest drugs given emergency approval by the United States Food and Drug Administration was Paxlovid (Burki, 2022). Qiu et al. revisited the usage of this drug and focused on drug administration beyond 5 days after the onset of symptoms. Their findings concluded that for administration beyond 5 days, Paxlovid has proven to be safe and effective in reducing viral load, without any additional adverse effects. Similarly, treatments like tocilizumab and baricitinib have both been used for treating COVID-19-triggered hyperinflammatory response. However, their safety and efficacy profiles have not been discussed. Zhang et al. performed a systematic review and meta-analysis comparing the efficacy and safety of tocilizumab and baricitinib in hospitalized COVID-19 patients. Both drugs showed similar efficacy in reducing 28-day mortality and hospital length of stay. However, baricitinib demonstrated a more favorable safety profile, with significantly lower rates of secondary infections, thrombotic events, bleeding events, and acute liver injury as compared to tocilizumab, making baricitinib a more attractive option for treating hospitalized COVID-19 patients.

Given that it is a constant race against time with these rapidly evolving viruses, the ability to repurpose existing drugs is crucial. Li et al. assessed the chemopreventive effect of ursodeoxycholic acid (UDCA) against COVID-19, relating to infection risk factors, symptoms, and recovery in outpatients with UDCA exposure. UDCA is an endogenous bile acid commonly used to treat various hepatobiliary diseases (Song et al., 2023) but it has also been shown to downregulate the ACE2 receptor used by SARS-CoV-2 for entry (Brevini et al., 2023). In their study, the UDCA-administered group demonstrated a lower infection rate as compared to their non-administered counterpart, while at the same time, mitigating the severity of the symptoms without any additional severe adverse effects.

Another therapeutic, lentinan, was found to exhibit antiviral and immunomodulatory effects. Administering lentinan, a β-glucan, as a nasal drop, Fan et al. evaluated its safety and efficacy as a COVID-19 treatment by performing a phase 1 dose-climbing study followed by a phase 2 randomized placebo-controlled study. The authors demonstrated that lentinan nasal drops were well-tolerated, reducing virus clearance and shedding time despite no significant difference in ameliorating symptoms. With lentinan being able to trigger the production of neutralizing antibodies, this treatment paves the way for the production of mucosal vaccines to generate mucosal immune responses against respiratory pathogens.

The advancement of new therapeutics to combat these elusive pathogens looks promising. However, the generalization of findings is currently limited by small cohort sizes and restricted geographical representation. Yet, the positive results should not be overlooked as it serves as a stepping stone for future discoveries. With the spotlight once again on the field of infectious diseases, it is only a matter of time before more prominent breakthroughs are achieved.
